# Sedentary behavior and early childhood health: associations between screen exposure, body mass index, and foot arch structure in preschool children

**DOI:** 10.3389/fpubh.2026.1858773

**Published:** 2026-07-06

**Authors:** Munise Duran

**Affiliations:** Department of Early Childhood Education, İnönü University, Malatya, Türkiye

**Keywords:** body mass index, child health, early childhood health, foot arch structure, physical development, screen exposure, sedentary behavior

## Abstract

Early childhood is a critical developmental period during which behavioral and anthropometric characteristics associated with child health begin to emerge. In recent years, screen exposure and sedentary behaviors have attracted increasing attention because of their potential associations with physical activity patterns, growth indicators, and musculoskeletal health outcomes in children. However, their relationships with foot arch categories during early childhood remain insufficiently explored. This analytical cross-sectional study aimed to examine the associations between screen exposure, body mass index (BMI) percentile, and foot arch categories in preschool children. The study included 509 children aged 5–6 years attending four public preschools in Çanakkale, Türkiye. Foot arch profile was assessed using a podoscope and classified according to the Staheli Arch Index and Jack test. Screen exposure was evaluated based on parent-reported daily duration categories, and BMI percentiles were calculated using World Health Organization reference standards. Multinomial logistic regression analysis was conducted to examine associations between screen exposure, BMI percentile, and foot arch categories after adjustment for age and sex. The findings indicated that both screen exposure and BMI percentile were significantly associated with specific foot arch categories. More consistent associations were observed particularly in the rigid flatfoot category, where higher screen exposure and higher BMI percentile were more frequently observed. However, the relatively small number of participants in the highest screen exposure category should be considered when interpreting these findings. Overall, the findings suggest that screen exposure and BMI percentile may be associated with specific foot arch categories during early childhood. Although causal interpretations cannot be made due to the cross-sectional design, the findings may contribute to the growing literature examining behavioral and anthropometric characteristics associated with musculoskeletal indicators in preschool children. Further longitudinal and intervention-based studies are needed to clarify the direction and underlying mechanisms of these associations.

## 1 Introduction

Early childhood is widely recognized as an important developmental period during which many behavioral and health-related patterns begin to emerge. Behavioral characteristics and physical indicators observed during this stage have been associated with later health outcomes and developmental processes (1, 2). From this perspective, early childhood represents an important period for research focusing on child health and development. Furthermore, the Developmental Origins of Health and Disease (DOHaD) framework emphasizes that early-life environmental and behavioral exposures may be associated with later health outcomes (3).

In recent years, increasing screen exposure among children has attracted growing attention in child health research. In particular, screen-based activities have been examined in relation to physical activity patterns and several health-related indicators in early childhood. International guidelines recommend limiting excessive screen exposure and supporting balanced movement behaviors during early childhood (4). However, global evidence suggests that many preschool-aged children exceed recommended screen exposure levels and do not consistently meet physical activity recommendations (5, 6). These findings highlight the growing interest in understanding behavioral characteristics associated with child health during early childhood.

The musculoskeletal system undergoes substantial developmental changes during early childhood. Within this context, foot arch profile has been examined in relation to lower limb alignment, plantar load distribution, and postural characteristics in pediatric populations (7, 8). Variations in foot arch profile are common during early childhood, and previous studies have reported associations between different foot arch categories, pes planovalgus morphology, and various anthropometric and functional characteristic (9, 40). Therefore, examining foot arch categories in preschool children may contribute to understanding musculoskeletal characteristics observed during this developmental period.

Previous research on foot arch profile has primarily focused on anthropometric and biomechanical characteristics, including body mass index (BMI), plantar pressure distribution, and pes planovalgus morphology (7, 10, 39, 40). More recently, behavioral and environmental characteristics have also attracted attention in studies examining child health and musculoskeletal outcomes. Ecological systems theory (11) emphasizes that child development occurs within multiple interacting environmental contexts, providing a broader framework for examining behavioral characteristics alongside physical health indicators during early childhood.

Sedentary behavior and screen exposure have been associated with several health-related and musculoskeletal outcomes in children in previous research (12, 13). However, the relationship between screen exposure and foot arch categories in preschool children remains insufficiently explored. This gap highlights the need for further research examining associations between behavioral characteristics and foot arch profiles during early childhood.

From a public health perspective, screen exposure and movement-related behaviors during early childhood have attracted increasing attention because of their potential associations with various health indicators. Previous studies have reported associations between screen exposure, physical activity patterns, obesity, and musculoskeletal outcomes in children (5, 6, 14). In addition, international guidelines recommend limiting excessive screen exposure and supporting balanced movement behaviors during early childhood (4, 15, 16).

In this context, examining foot arch categories together with screen exposure and BMI percentile may contribute to a broader understanding of behavioral and anthropometric characteristics observed during early childhood. Investigating these associations within preschool populations may also contribute to the literature on child health and musculoskeletal characteristics during this developmental period.

Accordingly, the present study aims to examine the associations between screen exposure, BMI percentile, and foot arch categories in preschool children. Using an analytical cross-sectional design, the study seeks to contribute to the literature by investigating associations between behavioral and anthropometric characteristics and foot arch profiles during early childhood.

## 2 Materials and methods

### 2.1 Study design

This study was conducted using an analytical cross-sectional design within the framework of quantitative research methods. Analytical cross-sectional studies are observational research designs that allow the examination of relationships between variables measured at a specific point in time (17, 18). This design is widely used in health research to evaluate associations between potential risk factors and health-related indicators.

In the present study, the relationships between foot arch structure, screen exposure, and body mass index percentile were examined among preschool children. In line with the analytical cross-sectional approach, all variables were assessed during the same time period, and variables associated with foot arch structure were examined using multivariable analyses. During this process, the potential confounding effects of age and sex were statistically controlled.

Because cross-sectional research designs do not establish temporal sequencing between variables, they do not permit causal inferences. Nevertheless, such designs provide an important methodological framework for identifying relationships between variables, describing potential patterns, and generating hypotheses for future longitudinal research (17, 19).

### 2.2 Participants and sampling

Ethical approval for this study was obtained from the Health Sciences Ethics Committee of Çanakkale Onsekiz Mart University (Decision No: 21/21, dated 28 May 2025). The ethical approval was granted within the scope of a broader research project that also included additional variables (e.g., postural assessments); however, the present study focused only on the variables relevant to the current research aims.

Data collection was carried out over a one-week period in June 2025 in four public preschools located in the city center of Çanakkale. Each preschool was visited for two consecutive days, and efforts were made to reach all eligible participants during this period under standardized assessment conditions.

The preschools included in the study were selected to reflect different socioeconomic characteristics within the city center of Çanakkale. During school selection, attention was paid to including public preschools from different residential areas with varying socioeconomic and sociocultural characteristics. However, individual-level socioeconomic status was not directly measured. Because school participation depended on institutional accessibility, administrative permission, and parental consent, the final recruitment process was based on convenience sampling, and no formal randomization procedure was applied.

The required sample size was determined *a priori* through power analysis conducted before data collection. Based on a chi-square test of independence, a medium effect size (Cohen’s w = 0.30; (20)), an alpha level of 0.05, and a statistical power of 0.80 were assumed. Considering the four-category structure of foot arch classification and the five-category structure of screen exposure, the minimum required sample size was calculated as 193 participants. To account for potential data loss, the target sample size was set at a minimum of 210 participants. The initial sample size calculation was based on the original five-category screen exposure structure. In the final analyses, the 3 h/day and ≥4 h/day categories were combined into a single ≥3 h/day category to reduce sparse-cell effects and improve the stability of multinomial regression estimates.

Participants were recruited using a non-probability sampling approach, specifically convenience sampling. All children enrolled in the selected preschools were invited to participate, and children whose parents provided written informed consent were included in the study. No randomization procedure was applied.

Initially, 530 children were assessed for eligibility. Of these, 12 children were excluded due to missing height and/or weight measurements, and 9 children were excluded because of incomplete screen exposure information. The final sample consisted of 509 children aged 5–6 years.

The final sample size exceeded the planned minimum sample size, which increased the statistical power of the analyses and improved the stability of estimates obtained from multinomial analyses involving multi-category variables. However, the relatively small number of participants in some screen exposure categories, particularly the higher screen exposure categories, should be considered when interpreting the findings.

### 2.3 Inclusion and exclusion criteria

Children aged 5–6 years whose parents provided written informed consent were included in the study. Participant eligibility was evaluated based on parental reports and school records.

Children with known neurological, orthopedic, or systemic conditions, congenital lower extremity deformities, or a history of foot surgery were excluded from the study. These exclusion criteria were applied to minimize the potential influence of clinical conditions that could affect foot arch structure.

Following the data collection process, children with missing height and/or weight measurements and those with incomplete screen exposure information were excluded from the final analyses. Accordingly, 12 children were excluded due to missing anthropometric data and 9 children were excluded because of incomplete screen exposure information.

### 2.4 Assessment of foot arch profile

Foot arch profile was assessed under standardized conditions using a podoscope. This assessment was based on weight-bearing plantar footprints and therefore provided an indirect, footprint-based estimate of the medial longitudinal arch profile rather than a direct anatomical evaluation of the bony or ligamentous arch structure. Measurements were conducted while children stood barefoot in a relaxed position with equal weight distributed on both feet.

**Figure 1:**
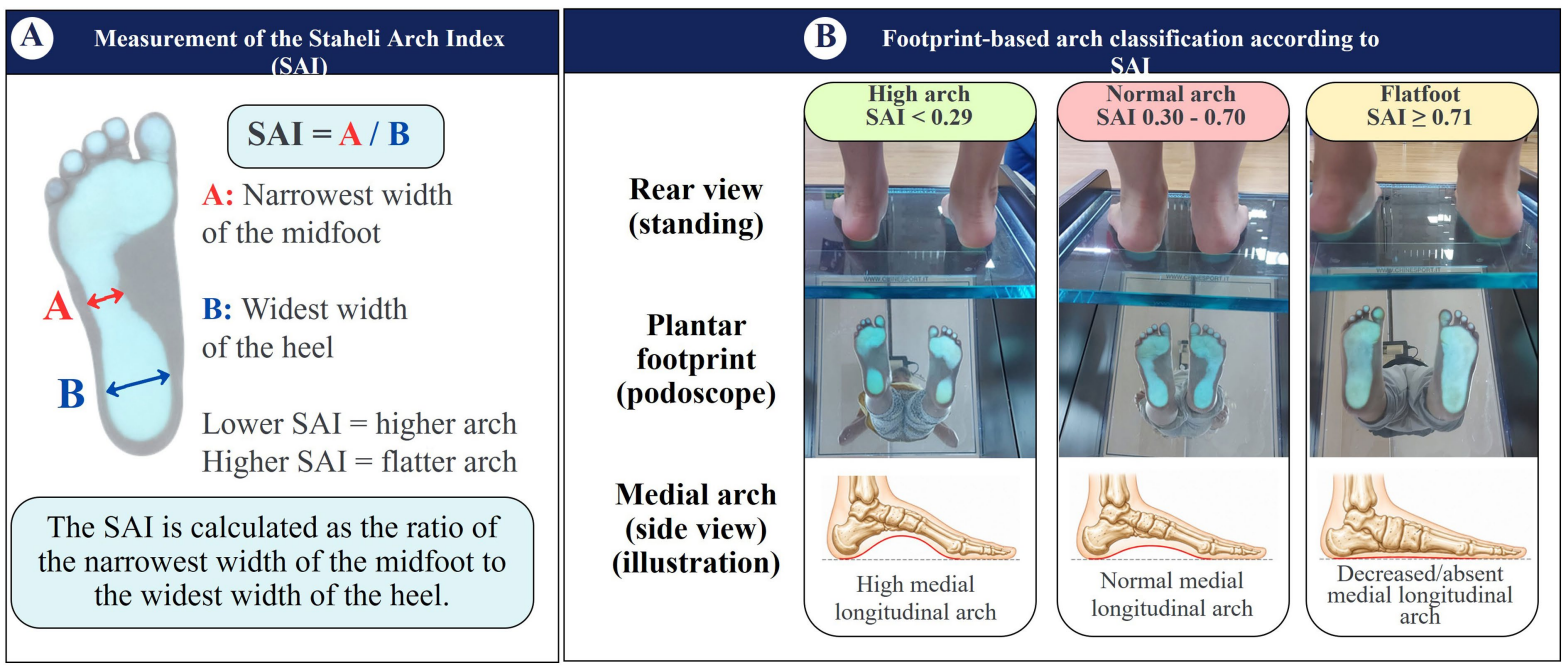
Stepwise footprint-based foot arch classification procedure using the staheli arch index (SAI). **(A)** Illustrates the calculation of the SAI as the ratio of the narrowest width of the midfoot to the widest width of the heel. **(B)** Presents representative podoscope images and corresponding medial longitudinal arch profiles used for classification into high arch (SAI < 0.29), normal arch (SAI = 0.30–0.70), and flatfoot morphology (SAI ≥ 0.71). Participants classified as having flatfoot morphology were subsequently evaluated using the Jack test to distinguish flexible flatfoot from rigid flatfoot (see Figure 2).

**Figure 2:**
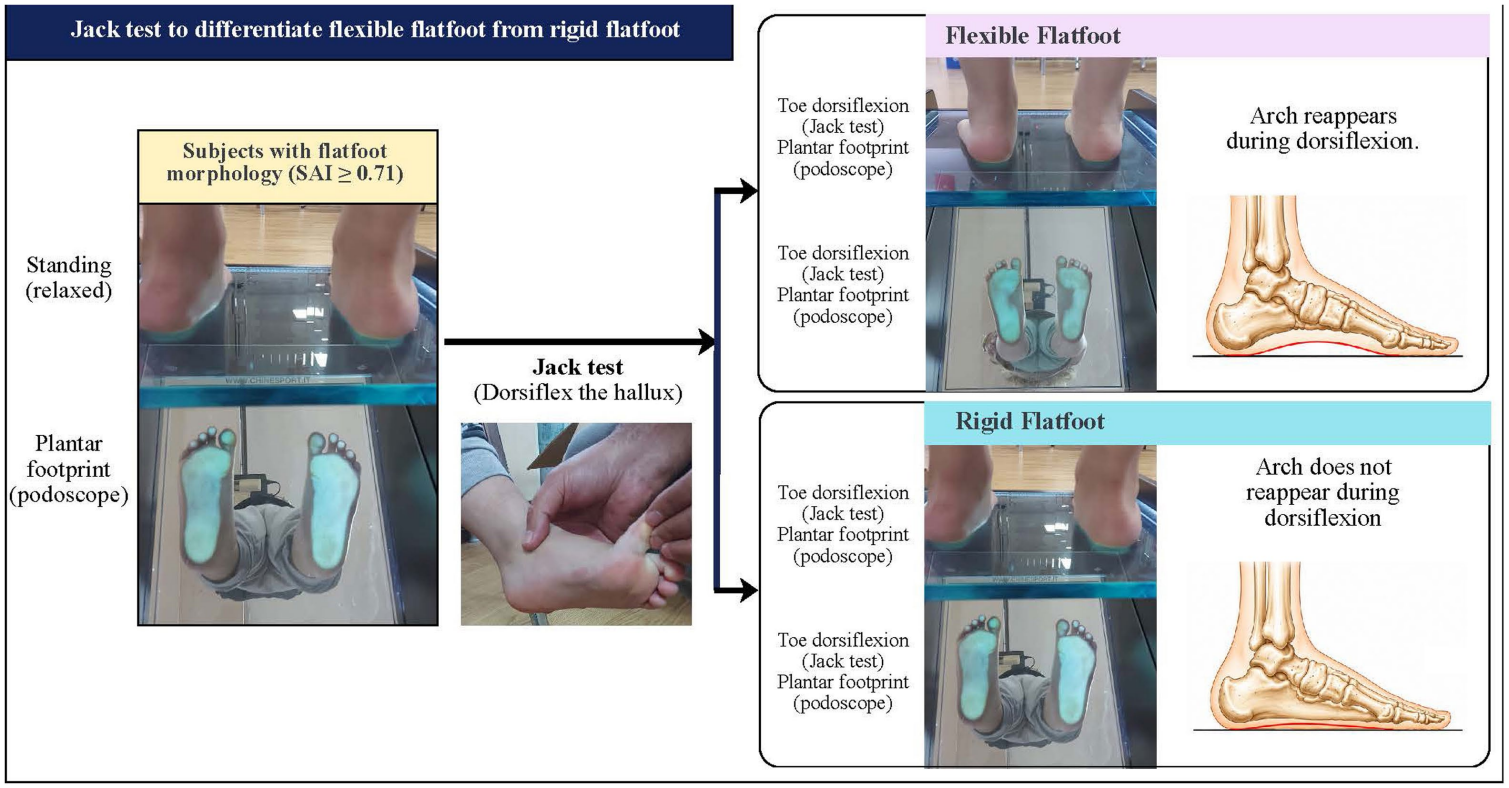
Differentiation of flexible and rigid flatfoot using the Jack test. Children initially classified as having flatfoot morphology (SAI ≥ 0.71) were further evaluated while standing. Reappearance of the medial longitudinal arch during hallux dorsiflexion was classified as flexible flatfoot, whereas absence of arch reconstitution was classified as rigid flatfoot. Representative podoscope images and schematic illustrations are shown for each condition.

Plantar footprints were recorded and evaluated using a stepwise classification procedure. A schematic representation of the footprint-based foot arch classification procedure according to the Staheli Arch Index (SAI), together with representative podoscope images for each category, is presented in Figures 1A,B. The Jack test assessment procedure used to distinguish flexible flatfoot from rigid flatfoot is illustrated in Figure 2.

First, the Staheli Arch Index (SAI), a widely used footprint-based measure for evaluating medial longitudinal arch characteristics in children, was calculated (21). The SAI was calculated as the ratio of the narrowest width of the midfoot to the widest width of the heel. In the present study, SAI values <0.29 were classified as high arch (pes cavus), values between 0.30 and 0.70 as normal arch, and values ≥0.71 as flatfoot morphology (7).

Second, plantar footprint patterns were visually reviewed to support the standardized classification procedure. A clearly defined medial arch concavity was interpreted as normal arch, an exaggerated concavity as high arch, and a reduced or absent medial arch concavity as flatfoot.

Third, children initially classified within the flatfoot morphology category were further evaluated using the Jack test (Hubscher maneuver) to distinguish flexible flatfoot from rigid flatfoot. During this test, passive dorsiflexion of the hallux was applied while the child was standing. Reappearance of the medial longitudinal arch during dorsiflexion was classified as flexible flatfoot, whereas absence of arch formation was classified as rigid flatfoot (9, 22, 23). Representative examples of the Jack test procedure are shown in Figure 2.

Based on this stepwise procedure, foot arch categories were assigned as normal arch, flexible flatfoot, rigid flatfoot, and high arch. All measurements and classifications were performed according to a standardized protocol by an experienced academic with prior expertise in footprint-based foot arch assessment procedures acquired through involvement in previously published observational studies (24, 25). To evaluate intra-rater reliability, 18 randomly selected plantar footprint images were reclassified by the same evaluator during a repeated evaluation process. Cohen’s kappa coefficient demonstrated excellent agreement between the two classifications (*κ* = 0.925, *p* < 0.001), indicating a high level of classification consistency. Previous studies have also reported that footprint-based measures, including the Staheli Arch Index, show good reliability in pediatric populations (7, 21).

### 2.5 Screen exposure

Screen exposure was assessed based on parental report. Parents were asked to report their child’s typical daily screen exposure during the previous 7 days, including television, tablet, and smartphone use combined across devices.

Rather than providing continuous estimates, parents selected from predefined screen-time categories (<1 h, 1 h, 2 h, and ≥3 h/day). This categorical approach was used to improve reporting consistency and standardize parental responses across participants.

Screen exposure was evaluated as a behavioral indicator potentially associated with sedentary lifestyle patterns rather than as a direct measure of physical inactivity.

### 2.6 Anthropometric measurements and BMI

Height and weight were measured using standard procedures, with children barefoot and wearing light clothing. Body mass index (BMI) was calculated and converted into age- and sex-specific percentile values based on World Health Organization growth reference standards (26).

BMI percentile was treated as a continuous variable in the analyses in order to preserve statistical power and avoid loss of information.

### 2.7 Statistical analysis

Statistical analyses were performed using IBM SPSS Statistics (Version 20). Descriptive statistics were calculated for all variables. Continuous variables were presented as mean ± standard deviation or median (interquartile range), depending on the distribution, while categorical variables were expressed as frequencies and percentages.

The chi-square test was used to examine associations between categorical variables, including foot arch structure, screen exposure, and sex. This test was applied as a preliminary analysis to identify potential relationships between variables.

Variables that showed significant associations in bivariate analyses, along with clinically relevant variables (age and BMI percentile), were included in a multinomial logistic regression model to evaluate the independent predictors of foot arch structure. The normal arch category was used as the reference group for the dependent variable. In the multinomial logistic regression analysis, the 1 h/day screen exposure category was used as the reference group for screen exposure comparisons. Adjusted odds ratios (ORs) and 95% confidence intervals (CIs) were reported.

This combined analytical approach, involving both bivariate (chi-square) and multivariable (multinomial logistic regression) analyses, is widely used in cross-sectional studies to examine associations between variables and to control for potential confounding effects (18, 19). A *p*-value of < 0.05 was considered statistically significant.

## 3 Results

### 3.1 Participant characteristics

A total of 509 children aged 5–6 years were included in the study. Of these, 75.8% were 6 years old and 53.8% were boys. The most common foot arch category was normal arch (58.7%), followed by rigid flatfoot (16.7%), flexible flatfoot (15.7%), and high arch (8.8%).

Regarding daily screen exposure, the largest proportions of children were in the 1-h/day (37.5%) and 2-h/day (31.0%) categories. In contrast, 15.3% of the children were included in the ≥3-h/day screen exposure category, while 16.1% were exposed to screens for less than 1 h/day. Detailed characteristics of the study participants are presented in Table 1.

**Table 1:** Characteristics of the study participants (*N* = 509).

Variable	Category	*n*	(%)
Age	5 years	123	24.2
	6 years	386	75.8
Sex	Female	235	46.2
	Male	274	53.8
Foot arch structure	Normal	299	58.7
	Flexible flatfoot	80	15.7
	Rigid flatfoot	85	16.7
	High arch	45	8.8
Screen exposure	<1 h	82	16.1
	1 h	191	37.5
	2 h	158	31.0
	≥3 h	78	15.3

### 3.2 Associations between screen exposure and foot arch structure

The distribution of screen exposure differed significantly across foot arch categories (*p* < 0.001). Children in the normal arch category were predominantly represented in the 1-h/day and 2-h/day screen exposure categories, whereas children with rigid flatfoot showed relatively higher proportions in the ≥3-h/day exposure category. Specifically, 49.4% of children with rigid flatfoot were exposed to screens for ≥3 h/day, compared with only 3.3% of children in the normal arch category.

**Figure 3:**
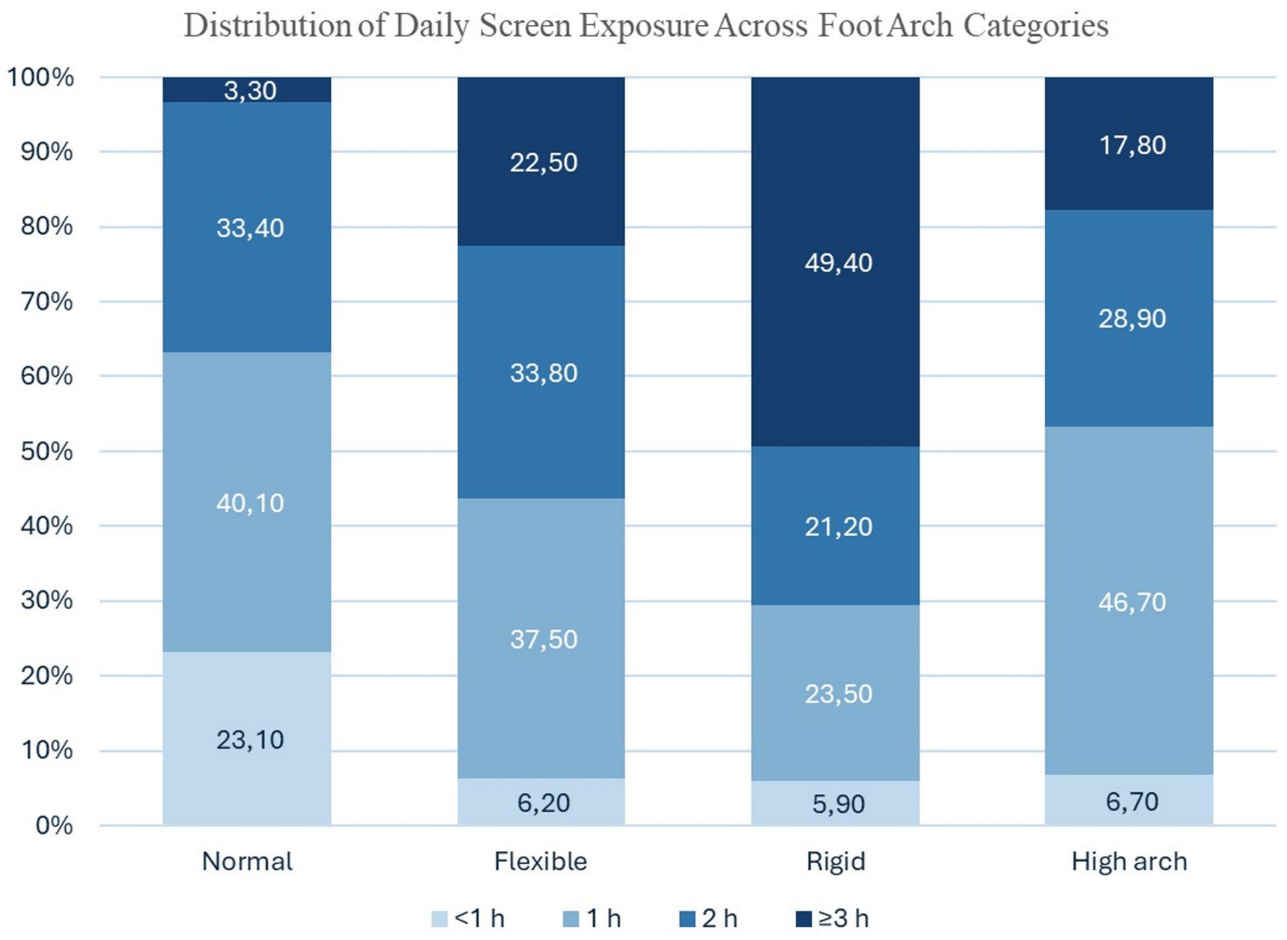
Distribution of daily screen exposure across foot arch categories. Stacked bar charts illustrate the percentage distribution of children with different levels of daily screen exposure (<1 h, 1 h, 2 h, and ≥3 h) within each foot arch category. The rigid flatfoot group showed the highest proportion of children with screen exposure ≥3 h per day, whereas the normal arch group demonstrated the lowest proportion in this category. Percentages are presented within each foot arch category.

Flexible flatfoot and high arch categories also demonstrated variations in screen exposure patterns; however, these distributions appeared less consistent than those observed for rigid flatfoot. The distribution of screen exposure across foot arch categories is presented in Table 2 and Figure 3. As illustrated in Figure 3, children with rigid flatfoot demonstrated relatively higher proportions in the ≥3-h/day screen exposure category compared with the other foot arch categories.

**Table 2:** Distribution of screen exposure across foot arch structure.

Foot arch category	<1 h *n* (%)	1 h *n* (%)	2 h *n* (%)	≥3 h n (%)
Normal arch	69 (23.1)	120 (40.1)	100 (33.4)	10 (3.3)
Flexible flatfoot	5 (6.2)	30 (37.5)	27 (33.8)	18 (22.5)
Rigid flatfoot	5 (5.9)	20 (23.5)	18 (21.2)	42 (49.4)
High arch	3 (6.7)	21 (46.7)	13 (28.9)	8 (17.8)
Total	82	191	158	78

Percentages represent row percentages within each foot arch category. Pearson chi-square test: *χ*^2^ = 126.624, df = 9, *p* < 0.001. No expected cell count was below 5.

### 3.3 Multinomial logistic regression analysis

Multinomial logistic regression analysis showed that screen exposure and BMI percentile were independently associated with specific foot arch categories after adjustment for age and sex (Table 3).

**Table 3:** Multinomial logistic regression analysis of factors associated with foot arch structure in preschool children (reference category: normal arch).

Variables	Flexible flatfoot OR (95% CI)	*p*	Rigid flatfoot OR (95% CI)	*p*	High arch OR (95% CI)	*p*
BMI percentile	1.00 (0.99–1.01)	0.585	1.02 (1.01–1.03)	0.001	0.99 (0.98–1.00)	0.025
Sex						
Male	0.76 (0.45–1.28)	0.306	0.36 (0.19–0.66)	0.001	1.25 (0.65–2.41)	0.497
Female (reference)	Reference		Reference		Reference	
Age						
5 years	0.41 (0.21–0.81)	0.009	0.82 (0.43–1.56)	0.547	0.30 (0.12–0.77)	0.012
6 years (reference)	Reference		Reference		Reference	
Screen exposure						
<1 h/day	0.25 (0.09–0.67)	0.006	0.42 (0.15–1.19)	0.102	0.23 (0.06–0.80)	0.021
2 h/day	1.09 (0.60–1.96)	0.780	1.10 (0.54–2.23)	0.794	0.77 (0.36–1.63)	0.495
≥3 h/day	7.97 (3.27–19.46)	<0.001	23.44 (9.87–55.67)	<0.001	6.23 (2.12–18.30)	0.001
1 h/day (reference)	Reference		Reference		Reference	

Multinomial logistic regression analysis examining predictors of foot arch structure. Reference categories: normal arch structure for the dependent variable; female for sex; 6 years for age; and 1 h/day for screen exposure. Odds ratios (ORs) and 95% confidence intervals (CIs) are reported.

The most consistent pattern was observed for rigid flatfoot. Compared with children in the 1-h/day screen exposure category (reference group), children exposed to screens for ≥3 h/day had significantly higher odds of rigid flatfoot (OR = 23.44, 95% CI: 9.87–55.67, *p* < 0.001). Higher BMI percentile was also associated with increased odds of rigid flatfoot (OR = 1.02, 95% CI: 1.01–1.03, *p* = 0.001).

For flexible flatfoot, children in the ≥3-h/day screen exposure category also demonstrated higher odds compared with the reference group (OR = 7.97, 95% CI: 3.27–19.46, p < 0.001). However, associations observed in some other screen exposure categories were smaller and less consistent.

Associations involving the high arch category were comparatively less consistent. Although children exposed to screens for ≥3 h/day demonstrated higher odds of high arch (OR = 6.23, 95% CI: 2.12–18.30, p = 0.001), these findings should still be interpreted cautiously.

Male sex was associated with lower odds of rigid flatfoot compared with females (OR = 0.36, 95% CI: 0.19–0.66, p = 0.001). In addition, children aged 5 years had lower odds of flexible flatfoot (OR = 0.41, 95% CI: 0.21–0.81, *p* = 0.009) and high arch (OR = 0.30, 95% CI: 0.12–0.77, *p* = 0.012) compared with children aged 6 years.

However, some estimates should still be interpreted cautiously due to the relatively small number of participants in the higher screen exposure categories.

## 4 Discussion

Early childhood represents an important period for musculoskeletal development and changes in foot arch profile, as well as a stage during which various health-related behaviors emerge. Previous studies have reported associations between biological maturation, behavioral characteristics, environmental conditions, and musculoskeletal indicators observed during this developmental period (7, 27). In addition, sedentary behaviors, particularly screen exposure, have been associated with physical activity levels, motor development, and several health-related indicators in early childhood (14, 28).

Recent biomechanical evidence suggests that sedentary behavior and reduced physical activity may be associated with musculoskeletal development through several pathways, including changes in muscular strength, flexibility, plantar loading, and gait-related biomechanics (29, 30). Lower physical activity levels have been associated with higher plantar pressures, longer contact times, and altered loading patterns during gait in children (30). In addition, overweight and obese children have been reported to demonstrate increased plantar loading, altered pressure distribution, and flatter foot structures compared with normal-weight peers (31). More recent studies have also suggested that asymmetrical plantar loading patterns and altered center-of-pressure trajectories may be related to differences in foot biomechanics and dynamic support of the medial longitudinal arch during childhood movement tasks (32). Although physical activity, gait mechanics, muscle function, and plantar pressure parameters were not directly assessed in the present study, these biomechanical observations may provide a possible contextual framework for interpreting the observed associations between screen exposure and foot arch characteristics.

The findings of the present study showed that both BMI percentile and screen exposure were significantly associated with specific foot arch categories when age and sex were controlled for. More consistent associations were observed particularly in the rigid flatfoot category compared with the other foot arch categories. These findings suggest that foot arch profile may be associated with anthropometric and behavioral characteristics assessed during early childhood. However, due to the cross-sectional design of the study, the observed associations should not be interpreted as causal or temporal relationships.

In the present study, higher screen exposure categories were more frequently observed among children with rigid flatfoot. More recent studies have also reported associations between screen exposure and musculoskeletal health outcomes in children and adolescents, although the underlying mechanisms remain unclear (13, 33). Some studies have also discussed reduced mechanical loading as a possible explanation for musculoskeletal differences observed in relation to sedentary lifestyles (34). However, physical activity levels and mechanical loading were not directly assessed in the present study. Therefore, such explanations should be considered speculative and interpreted cautiously.

In addition, the relationship between sedentary behavior and BMI has been widely discussed in the public health literature. Previous studies have reported associations between screen exposure and higher BMI levels in children (35). In the present study, higher BMI percentiles were particularly associated with the rigid flatfoot category. Similar associations between body weight–related measures and foot structure have also been reported in previous pediatric studies (9, 36). More recent evidence has likewise emphasized that the relationship between body mass and pediatric flatfoot remains complex and may vary depending on the assessment methods, age groups, and morphological characteristics evaluated (37, 38). For example, Kopaczyńska et al. (38) reported that increased BMI was not consistently associated with foot structure abnormalities in preschool children. However, because behavioral variables such as physical activity were not directly assessed in the present study, the mechanisms underlying these observed associations remain unclear.

The coexistence of higher BMI percentile and higher screen exposure observed in some foot arch categories may represent an observable pattern in early childhood; however, the underlying mechanisms of these associations remain unclear. Due to the cross-sectional design, the direction of these relationships cannot be established, and the possibility of reverse causality should also be considered. For example, differences in foot structure may be associated with variations in children’s movement patterns or activity preferences, which could also relate to screen exposure behaviors.

From a public health perspective, the present findings indicate that screen exposure and BMI percentile may be associated with certain foot arch categories in early childhood. Although the underlying mechanisms and temporal direction of these relationships remain unclear, the findings may contribute to the broader literature examining sedentary behaviors and health-related characteristics during the preschool period. The observed associations are also broadly consistent with current international guidelines recommending the limitation of screen exposure and the support of healthy movement behaviors in early childhood (4, 15).

In this context, the present study contributes to the literature by examining associations between screen exposure, BMI percentile, and specific foot arch categories in preschool children within a cross-sectional framework. The findings may contribute to the growing body of research investigating sedentary behaviors and musculoskeletal characteristics during early childhood. Future research using longitudinal and intervention-based designs is needed to clarify the temporal direction and potential explanations underlying these observed associations.

### 4.1 Implications for practice and public health

The findings of the present study suggest that screen exposure and BMI percentile may be associated with specific foot arch categories in preschool children. Although the underlying mechanisms and temporal direction of these associations remain unclear, the findings may contribute to the broader literature examining sedentary behaviors and musculoskeletal characteristics during early childhood.

From a public health perspective, the observed associations highlight the importance of monitoring health-related behaviors during the preschool period. The findings are also broadly consistent with current international guidelines emphasizing balanced movement behaviors and the limitation of excessive screen exposure in early childhood (4, 15, 16).

However, because physical activity, motor development, and mechanical loading were not directly assessed in the present study, potential explanations regarding these associations should be interpreted cautiously. Further longitudinal and intervention-based research is needed to clarify the temporal direction and possible mechanisms underlying these observed relationships.

### 4.2 Strengths and limitations

This study has several strengths. First, it was conducted with a relatively large sample of preschool children, which enhances the potential of the findings to reflect behavioral and anthropometric patterns observed during early childhood. Second, foot arch categories were examined using a multi-category classification approach rather than a binary categorization, allowing for a more nuanced assessment of variations in foot arch profile. In addition, the combined evaluation of screen exposure and BMI percentile provides a broader perspective on behavioral and anthropometric characteristics associated with foot arch categories in early childhood.

However, several limitations should be considered when interpreting the findings. First, due to the cross-sectional design, the observed associations cannot be interpreted as causal relationships. Accordingly, the findings reflect associations between variables rather than cause-and-effect mechanisms. In addition, screen exposure was assessed based on parental reports, which may be subject to recall bias.

Important potential confounding variables, such as physical activity levels and socioeconomic status, were not directly measured in this study. This limitation should be taken into account when interpreting the observed associations between screen exposure and foot arch categories. Furthermore, although statistical adjustments were made for age and sex, residual confounding cannot be completely ruled out.

Another limitation is the relatively small number of children in the higher screen exposure categories, which may have affected the stability and precision of some regression estimates despite the reorganization of screen exposure groups to reduce sparse cell effects. In terms of measurement, foot arch profile was assessed using a static footprint-based method, and functional evaluations such as dynamic gait analysis were beyond the scope of this study. Although intra-rater reliability demonstrated excellent agreement, inter-rater reliability was not assessed because all evaluations were performed by a single evaluator.

Finally, the study was conducted using a convenience sampling method in a limited number of public preschools within a single province, which may restrict the generalizability of the findings. In addition, the possibility of reverse causality cannot be excluded due to the cross-sectional design. These limitations should be carefully considered when interpreting the results.

## 5 Conclusion

The present study demonstrated significant associations between screen exposure, BMI percentile, and specific foot arch categories in preschool children. More consistent associations were observed particularly in the rigid flatfoot category. These findings contribute to the growing body of literature examining behavioral and anthropometric characteristics associated with foot arch profiles during early childhood.

However, because of the cross-sectional design, the observed associations should not be interpreted as causal or temporal relationships. In addition, physical activity, motor development, and mechanical loading were not directly assessed in the present study; therefore, potential explanations regarding these associations should be interpreted cautiously.

Further longitudinal and intervention-based studies are needed to clarify the temporal direction and possible mechanisms underlying these observed relationships.

## Data Availability

The raw data supporting the conclusions of this article will be made available by the authors, without undue reservation.
